# Retraction: Short communication: Upregulation of hypoxia/reoxygenation-induced Shc3 by downregulated miR-455-5p, suppresses trophoblast invasion and is associated with placental inflammation and angiogenesis in preeclampsia

**DOI:** 10.1371/journal.pone.0344272

**Published:** 2026-03-05

**Authors:** The PLOS One Editors

Following the publication of this article [1], concerns were raised about Fig 1. Specifically, there appears to be overlap between panels in Fig 1 in [1], and between the original images in [2], representing different experimental conditions:

- In Fig 1C, between the panels
  - Invasion (60h) Control and Invasion (60h) miR-455-5p mimic
  - Invasion (60h) Control and Invasion (60h) miR-455-5p mimic-NC
  - Invasion (60h) miR-455-5p mimic and Invasion (60h) miR-455-5p mimic-NC
  - Migration (18h) Control and Migration (18h) miR-455-5p mimic
- In Figure-1_C.zip [2] between original images
  - invasion-hypoxia-mimic(3).png and invasion-hypoxia-Control(3).png
  - In Figure-1_D.zip [2] between original images
  - invasion-Normoxia-Nc(1).png and invasion-Normoxia-mimic(3).png
  - invasion-Normoxia-Nc(3).png and invasion-Normoxia-mimic(2).png
  - invasion-Normoxia-Nc(3).png and invasion-Normoxia-Control(2).png
  - invasion-Normoxia-mimic(2).png and invasion-Normoxia-Control(2).png
  - Normoxia-mimic(2).png and Normoxia-Control(3).png

In light of the above unresolved concerns that question the reliability and integrity of the results presented in [1], the *PLOS One* Editors retract this article.

All authors did not agree with the retraction.
